# Preferential transfer of mitochondria from endothelial to cancer cells through tunneling nanotubes modulates chemoresistance

**DOI:** 10.1186/1479-5876-11-94

**Published:** 2013-04-10

**Authors:** Jennifer Pasquier, Bella S Guerrouahen, Hamda Al Thawadi, Pegah Ghiabi, Mahtab Maleki, Nadine Abu-Kaoud, Arthur Jacob, Massoud Mirshahi, Ludovic Galas, Shahin Rafii, Frank Le Foll, Arash Rafii

**Affiliations:** 1 Stem Cell and Microenvironment Laboratory, Weill Cornell Medical College in Qatar, Education City, Qatar Foundation, Doha PO: 24144, Qatar; 2 Department of Genetic Medicine, Weill Cornell Medical College, New York, NY, USA; 3 UMRS 872 INSERM, Université Pierre et Marie Curie-Paris 6 and Université Paris Descartes, Equipe 18, Centre de Recherche des Cordeliers, 15 rue de l’Ecole de Medecine, Paris Cedex 06 75270, France; 4 Primacen Cellular Imaging Platform, University of Rouen, Mont-Saint-Aignan 76821, France; 5 Laboratory of Ecotoxicology, University of Le Havre, Le Havre 76058, France

**Keywords:** Tunneling nanotubes, Intercellular transfer, Endothelial cells, Cancer cells, Chemoresistance

## Abstract

Our vision of cancer has changed during the past decades. Indeed tumors are now perceived as complex entities where tumoral and stromal components interact closely. Among the different elements of tumor stroma the cellular component play a primordial role. Bone Marrow derived mesenchymal cells (MSCs) are attracted to tumor sites and support tumor growth. Endothelial cells (ECs) play a major role in angiogenesis. While the literature documents many aspects of the cross talk between stromal and cancer cells, the role of direct hetero-cellular contact is not clearly established. Recently, Tunneling nanotubes (TnTs) have been shown to support cell-to-cell transfers of plasma membrane components, cytosolic molecules and organelles within cell lines. Herein, we have investigated the formation of heterocellular TnTs between stromal (MSCs and ECs) and cancer cells. We demonstrate that TnTs occur between different cancer cells, stromal cells and cancer-stromal cell lines. We showed that TnTs-like structure occurred in 3D anchorage independent spheroids and also in tumor explant cultures. In our culture condition, TnTs formation occurred after large membrane adhesion. We showed that intercellular transfers of cytoplasmic content occurred similarly between cancer cells and MSCs or ECs, but we highlighted that the exchange of mitochondria occurred preferentially between endothelial cells and cancer cells. We illustrated that the cancer cells acquiring mitochondria displayed chemoresistance. Our results illustrate the perfusion-independent role of the endothelium by showing a direct endothelial to cancer cell mitochondrial exchange associated to phenotypic modulation. This supports another role of the endothelium in the constitution of the metastatic niche.

## Background

Tissues are comprised of different cell types that communicate to maintain homeostasis [1]. While cell number and positional identity of normal tissues are lost in cancers, tumor cells keep many interactions with surrounding non-malignant cells and extra-cellular matrix [2]. Indeed, primary and metastatic tumors contain a variety of stromal cell types such as endothelial cells (ECs), fibroblasts and various bone marrow-derived cells, including macrophages, mast cells, and mesenchymal stem cells (MSCs) [3]. These various cell types are recruited to the tumor site and through the modulation of different pathways contribute to tumor growth and metastasis [4-10]. The interaction between cancer and stromal cells are primordial for the biology of the tumors. Several lines of evidence have been provided. The cross talk between cancer cells and fibroblasts induces phenotypic modification of the stromal cells toward a Cancer Associated Fibroblasts (CAF) phenotype promoting in return the cancer cells [11]. In the metastatic context bone marrow derived cells can be attracted to the site of metastasis and prepare the metastatic niche [12]. Comforting these findings, Park group has demonstrated that a signature of the stroma can predict outcome in breast cancer patients [13]. Understanding the mechanisms implicated in the relationship between cancer cells and stromal cells are thus essential to target tumor progression.

Several mechanisms are involved in the cross talk between cancer and stromal cells: (i) cytokine/receptor interaction [8,14] (ii) direct cell contact and material exchange [15-17] (iii) micro-particle mediated cell communication [18]. Recently the role of contact-dependent interactions between cancer and stromal cells has been emphasized. These are mediated by synapses, GAP junction and cell-to-cell communication through tunneling nanotubes (TnTs) [19]. First described in cultured rat pheochromocytoma PC12 cells [20], TnTs have been observed in multiple cell types in vitro [21] and more recently in vivo [22,23]. These thin plasma membrane structures connecting cells allow intercellular transfers of organelles, various plasma membrane components and cytoplasmic molecules [24]; calcium ions, major-histo-compatibility proteins (MHC class I), pathogens, small organelles of the endosomal/lysosomal system, mitochondria and ATP-Binding Cassette transporter (P-glycoprotein) have all been shown to be transferred between different cells by TnTs [19,25-29]. While the importance of TnTs-mediated interaction in vivo is still debated in the literature due to lack of a specific inhibitor to tailor their role, there are now evidences demonstrating the functional role of TnTs-mediated intercellular transfer [30].

Several studies demonstrate the existence of TnTs mediated transfers between cancer cells [31]. Thus presence of TnTs was established using cells derived from patient specimens as well as from established cancer cell lines. Different components are transferred through TnTs. However the role of TnTs in forming direct cytoplasmic connections between cancer and stromal cells and their consequences remains to be established.

In this study, we hypothesized that TnTs could mediate cytoplasmic exchange and phenotype transfer between stromal and cancer cells. In our culture conditions we observed TnTs, established between different cancer cells (breast and ovarian cancer cell lines) and stromal cells (mesenchymal stem cells and endothelial cells). We described spontaneous exchange of cytoplasmic material between ECs or MSCs and multiple cancer cell lines. We showed a preferential transfer of mitochondria from endothelial to cancer cells resulting in acquisition of chemoresistance. Thus, our results raise the possibility of direct intercellular exchange as a modulator of tumor phenotype by the stromal component.

## Experimental procedures

### Cell culture

Ovarian cancer cells lines SKOV3 (HTB-77), OVCAR3 (HTB-161), breast cancer cell lines MDA-MB231 and MCF7 were purchased from the American Type Culture Collection (ATCC, Manassas, VA, USA). Cells were grown in DMEM high glucose (Hyclone, Thermo Scientific) supplemented with 20% FBS (Hyclone, Thermo Scientific), 1% Penicillin-Streptomycin-Amphotericyn B solution (Sigma), 2 mM L-glutamine (Sigma), 1X Non-Essential Amino-Acid (Hyclone, Thermo Scientific).

The E4ORF1^+^ ECs (E4^+^ECs) were generously provided by Shahin Rafii [32]. The cells were grown in M199 (Gibco, Life Technologies) supplemented with 20% FBS, 1% Penicillin-Streptomycin-Amphotericyn B solution (Sigma), 4 mM L-glutamine, 50 μg/ml heparin (Sigma) and 30 μg/ml β EC growth factor (βECGF, Upstate Biotechnology, Lake Placid, NY, USA). These cells express green fluorescence protein (GFP).

Mesenchymal stem cells (MSCs) were purchased from Stem Cells, Inc (MSC-001 F, Vancouver, CA) maintained and expanded in culture using MesenCult® MSC Basal Medium completed with Mesenchymal Stem Cell Stimulatory Supplements (Stem Cell Inc, Vancouver, CA). Their ability to differentiate in adipocytes, osteoblasts and chnodrocytes was verified as instructed by the supplier (data not shown). All cultured cells were incubated at 37°C under a water-saturated 95% air-5% CO_2_ atmosphere. Shaking cultures were performed similarly using a rocking plates at low speed.

### Sphere formation

Cells were dissociated into single cell suspension by trypsinization and further sieving through 40-um cell strainers and subsequently resuspended in 3D media containing DMEM F-12 supplemented with 2% B27 (Invitrogen), 20 ng/mL VEGF (Peprotech), 20 ng/mL bFGF (Peprotech), and 5 ug/mL insulin (Sigma). The cells were plated at 5000 cells per well of ultralow attachment 24-well plates (Costar, Corning) and were grown in a humidified incubator at 37°C and 5% CO_2_. The media was changed every second day. Primary spheres started to form at day 3 and continued to be cultured for up to 5 days.

### Explant culture

Tumor material from patients presenting advanced ovarian carcinoma Stage IIIC were included in this study (IRB Number: #9161/2010, “Isolation and characterization of cancer stem cells”). During debulking surgery metastatic nodules were removed and processed as follows. Upon serial washing with PBS and red blood cell Lysis buffer (eBiosciences, San Diego, USA) nodules were minced and cultured with or without feeders (EC^+^E4) in DMEM high glucose (Hyclone, Thermo Scientific) supplemented with 20% FBS (Hyclone, Thermo Scientific), 1% Penicillin-Streptomycin-Amphotericyn B solution (Sigma), 2 mM L-glutamine (Sigma), 1X Non-Essential Amino-Acid (Hyclone, Thermo Scientific).

### Labeling of cell organelles

To trace intercellular exchange of mitochondria, cells were separately labeled with MitoTracker DeepRed and Green (Invitrogen) according to the manufacturer’s protocol [26]. Briefly, cells were resuspended in prewarmed (37°C) staining solution containing the MitoTracker® probe (50 nM) for 30 minutes under growth conditions appropriate. After staining, cells were washed 3 times in PBS and resuspended in fresh prewarmed medium. Cells were plated in mono or co-culture according to the experiment.

### Confocal microscopy

Live-cell TnTs imaging using previously published protocols was performed [20]. Cells were labeled with 1 mg/ml Alexa Fluor® 594 conjugated wheat germ agglutinin (WGA, Invitrogen SARL, Cergy Pontoise, France) at 5 μg/ml for 10 minutes at 37°C in the dark. WGA is a probe for detecting glycoconjugates, which selectively bind to N-acetylglucosamine and N-acetylneuraminic acid residues of cell membranes. Imaging was performed using a Zeiss confocal Laser Scanning Microscope 710 (Carl Zeiss). Post-acquisition image analysis was performed with Zeiss LSM Image Browser Version 4.2.0.121 (Carl Zeiss).

### Microparticle purification

MPs from eGFP-E4^+^ECs were isolated as previously described [16]. Briefly, 48-h-supernatants of 80% serum-starved confluent tumor or endothelial cells were collected and sequentially centrifuged (4°C) at 300 g for 10 min, 800 g for 10 min, and at 3000 g for 15 min. MPs were pelleted at 100,000 g for 1 h, and washed once in PBS and centrifuged again at 100,000 g for 1 h. The final pellet containing purified MP was labeled with MitoTracker DeepRed to assess mitochondria transfer by microparticles.

### Analysis of transfer by flow cytometry

After co-culture, cells were trypsinized, resuspended and washed with PBS. Fluorescence (FL) was quantified on a SORP FACSAria2 (BD Biosciences). Data were processed with FACSDiva 6.3 software (BD Biosciences). Doublets were excluded by FSC-W x FSC-H and SSC-W x SSC-H analysis; eGFP fluorescence was acquired with 488 nm blue laser and 510/50 nm emission, Mitotracker Deep Red was acquired with 640 nm red laser and 670/14 nm emission. Figures display the median of fluorescence intensity (mfi) relative to control. Single stained channels were used for compensation and fluorophore minus one (FMO) controls were used for gating. 20,000 events were acquired per sample.

### Cell viability study (MTT assay)

Viability of cells was examined with an MTT assay. 24 hours after treatment with doxorubicin, 10% of MTT reagent was added to each well to a final concentration of 500 μg/ml, and the cells were incubated for 4 hours at 37°C. 100 μl of DMSO were added to each well. Optical density was read at 570 nm versus 630 with an EnVision multilabel reader (PerkinElmer, Massachusetts, USA). 3 triplicates were performed per condition.

### Statistical analysis

All quantitative data are expressed as mean ± standard error of the mean (SEM). Statistical analysis was performed using SigmaPlot 11 (Systat Software Inc., Chicago, IL). A Shapiro-Wilk normality test, with a p = 0.05 rejection value, was used to test normal distribution of data prior to further analysis. All pairwise multiple comparisons were performed by one way ANOVA followed by Holm-Sidak posthoc tests for data with normal distribution or by Kruskal-Wallis analysis of variance on ranks followed by Tukey posthoc tests, in case of failed normality test. Paired comparisons were performed by Student’s t-tests or by Mann–Whitney rank sum tests in case of unequal variance or failed normality test. Statistical significance was accepted for p < 0.05 (*), p < 0.01 (**) or p < 0.001 (***). All experiments were repeated at least three times.

## Results

### Homocellular and heterocellular TnTs occur spontaneously in various cancer cell cultures

**Figure 1:**
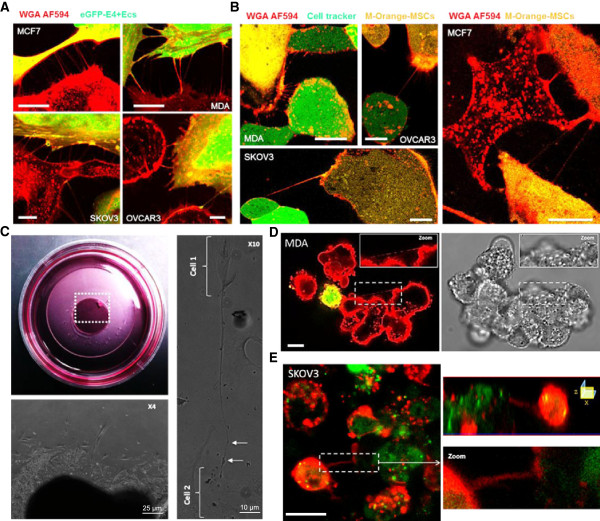
**Hetero**-**cellular Tunneling NanoTubes** (**TnTs) connect stromal to various tumor cells in culture. A**, confocal imaging of TnTs in co-culture system. Different tumoral cell lines were co-cultured with eGFP-E4^+^ECs. Before imaging by confocal microscopy, the co-cultures were stained with Alexa Fluor 594 conjugated-wheat germ agglutinin (WGA AF594) to reveal cell membranes. Hetero-cellular TnTs can be observed between eGFP-E4^+^ECs in each of the four cell lines studied (MCF7 *top left panel*, MDA *top right panel*, SKOV3 *bottom left panel*, OVCAR3 *bottom,right panel*). *Scale bar: 5 μm*. **B**, confocal imaging of TnTs in co-culture system. MDA, OVCAR3, SKOV3 or MCF7 cells were stained with CellTracker Green, mixed 1/1 with, M-orange-stained Mesenchymal Stem Cells (MSCs) and plated on glass coverslips for subsequent co-cultures. Before imaging, membranes of the co-cultured cells were stained with WGA AF594. Representative images of hetero-cellular TnTs connecting MDA, OVCAR3, SKOV3 or MCF7 cells TnTs to MSCs are presented. *Scale bar: 10 μm*. **C**, Phase contrast imaging of ovarian cancer explant. Explants of metastatic ovarian cancer nodules were cultured during ten days in Petri dishes (*top left panel*). Cells spreading from 3D explants (*bottom left panel*). TnTs-like structures with high length-to-diameter ratios bridged cells (*cell 1 to cell 2 in the right panel*). **D**, confocal imaging of 3D cultures. Angiospheres containing MDA and eGFP-E4^+^ECs were grown in 3D media for three days and stained with WGA AF594. TnT can be identified between cancer cells in fluorescence (*left panel*) and in transmission (*right panel*) in distinct sphere formations. *Scale bar: 10 μm*. **E**, confocal imaging of 3D cultures. Angiospheres containing SKOV3 and eGFP-E4^+^ECs were grown in 3D media for three days and stained with WGA AF594. TnT connecting cancer cells and eGFP-E4^+^ECs can be observed. Z-X reconstitution shows TnT typical no attachment to the substrate. *Scale bar: 10 μm*.

Previous studies reporting the occurrence of TnTs in vitro mainly focus on a specific cell types. Here we determined the presence of TnTs between various cancer and stromal cells under the same culture conditions. Four cancer cell lines were assessed (MCF7 and MDA derived from breast cancers, and SKOV3 and OVCAR3 derived from ovarian cancers). Homo-cellular TnTs were observed in all cancer cell lines studied (Additional file 1: Figure S1A). Similarly TnTs were also found in non-malignant E4^+^ECs and MSCs (Additional file 1: Figure S1B-C). To study the role of TnTs in interactions between stromal and cancer cells, M-Orange-MSCs and eGFP-E4^+^ECs were co-cultured with different cancer cells. TnTs were found connecting cancer cells and M-Orange-MSCs or eGFP-E4^+^EC (Figure 1A-B).

All observed structures displayed the specific characteristics of TnTs: (i) they had no contact to the substratum and hover in the extracellular medium (Additional file 1: Figure S2A to C, Additional file 2: Movie 1) (ii) they exhibited a typical width of less than 0.5 μm and a length over several cell diameters (Additional file 1: Figure S2D-E), (iii) they contained F-actin (Additional file 1: Figure S2F).

We then studied explants from ovarian cancer metastatic nodules to test whether TNTs could occur in a tumor context. We cultured these in a 3D setting and were able to visualize TnTs-like structures at the periphery of the explants (Figure 1C and Additional file 1: Figure S3A). Recently there has been an emphasis on other cell culture conditions such as spheroid formation assay. These are performed in low-adherent culture plates and consist in the formation of floating cellular aggregates that evolving in cell spheroids. We therefore assessed the occurrence of TnTs in a 3D anchorage independent spheroid co-culture system. Confocal microscopy imaging of three days-old mixed spheres (stromal-cancer cells) displayed TnTs between: (i) cancer cells, (ii) cancer and stromal cells (Figure 1D and E and Additional file 1: Figure S3B and C).

### Formation of TnTs

**Figure 2:**
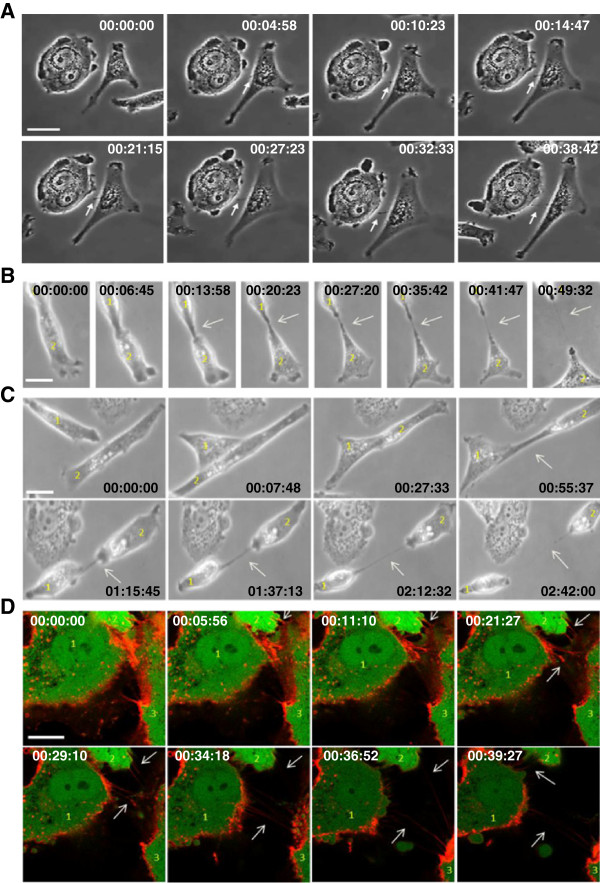
**Time-lapse studies of TnTs formation. A**, detailed frames extracted from time-lapse recording of the movie 2. This event happened at 43 seconds in the left corner of movie 2. Duration was reported, 38 min 42 second in total. This set of pictures allows us to see formation of a TnT by membrane projection between MCF-7 and MSCs co-cultured for 24 hours. *Scale bar: 10 μm*. **B**-**C**, detailed frames extracted from time-lapse recording of the movie 2. This event happened at 1 min 10 seconds in the left top corner (**B**) and at 1 min 12 seconds on the right top corner (**C**). Duration was reported, 49 min 32 seconds (B) and 2 hours 42 min (**C**). Serial images showing the formation of a nanotube following large membrane adhesion between MCF-7 and MSCs *Scale bar: 20 μm*. **D**, Detailed frames extracted from time-lapse recording of the movie 5. MCF-7 and eGFP-E4^+^ECs were co-cultured and stained using WGA AF594 to reveal cell membranes. Separated cells migrated toward each other. After the contact lasting the two cells formed nanotubes (arrows) elongating as the cells separated further*. Scale bar: 10 μm*.

Several models have been proposed for the formation of TnTs, mainly describing the connection between projections of two cell membranes [30,33]. We observed, in our culture conditions, such projections of TnTs between two cells (Figure 2A and Additional file 3: Movie 2). However, using time-lapse studies, we mainly observed the following succession of events: cell migration, large intercellular adhesion, cell separation, occurrence and/or persistence of TnTs, disruption of TnTs as the cells separated further (Figure 2B-D and Additional file 3: Movies 2, Additional file 4: Movie 3, Additional file 5: Movie 4 and Additional file 6: Movie 5). This emphasizes the role of large membrane adhesions before the formation of TnTs.

### TnTs mediate preferential transfer of cytoplasmic components from stromal cells to cancer cells

**Figure 3:**
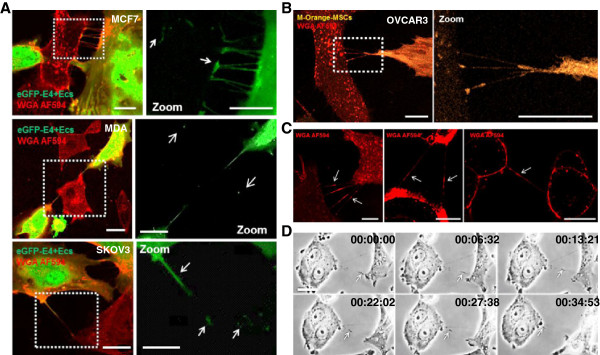
**Intercellular transfer of cytoplasmic contents through TnTs.** Tumoral cells were co-cultured with eGFP-E4^+^ECs or M-Orange-MSCs. Before imaging by confocal microscopy, the co-cultures were stained with WGA AF594 to reveal cell membranes. Confocal imaging showed hetero-cellular TnTs containing GFP (**A**) or M-Orange (**B**). WGA-stained cytoplasmic content were also seen at several points in TNTs (**C**, *arrows*). Subsequently GFP or M-Orange could be observed in recipient cancer cells in different panels. **A**, GFP dots (*arrows*) could be observed in recipient cells in different panels (MCF7 *on the top*, MDA *on the center*, SKOV3 *on the bottom*) *Scale bar: 10 μm*. **B**, M-orange could be detected in recipient OVCAR3 cancer cells. *Scale bar: 20 μm*. **C**, *Scale bar: 10 μm*. **D**, detail frames extracted from time-lapse recording of the movie 2. This event happens at 55 seconds in the left side of movie 2. Duration was reported, 34 min 53 second in total. Cytoplasmic material was transferred through TnTs as indicated (*arrow*). *Scale bar: 10 μm*.

**Figure 4:**
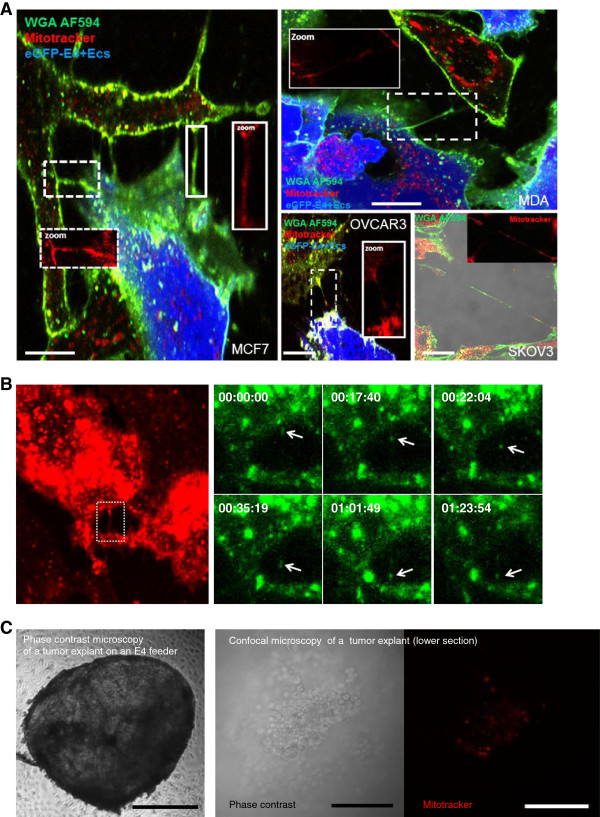
**Transfer of Mitochondria between eGFP-E4**^**+**^**ECs and cancer cells. A***,* confocal images of mitochondria transfer through TnTs. eGFP-E4^+^ECs were stained with MitoTracker Deep-Red to follow their mitochondria. Subsequently, they were co-cultured with cancer cells. Prior to confocal imaging, co-cultures were stained with WGA AF594 to reveal cell membranes. We identified transfer of mitochondria through TnTs from eGFP-E4^+^ECs to cancer cells (MCF7 *right panel*, MDA *left top panel*, OVCAR3 and SKOV3 *left bottom panels*). MitoTracker staining could also be observed in cancer cells cytoplasm. *Scale bar: 10 μm*. **B**, detailed frames extracted from movie 8, WGA staining shows the presence of a TnT (*left panel*), Mitotracker stained material transfer can be observed within the TnT (*right panel*) **C**, phase contrast imaging of ovarian cancer explant on eGFP-E4^+^ECs layer. eGFP-E4^+^ECs were stained with MitoTracker Deep-Red. Tumor explants were cultured on a feeder of eGFP-E4^+^ECs stained with Mitotracker (*Let panel, scale bar: 500 μm*). Confocal imaging were performed after 48 hours of culture. Mitochondria from eGFP-E4^+^ECs were observed within the tumor explant (*Middle and right panel, scale bar: 100 μm*).

We used co-culture experiments to investigate a potential exchange of cytosolic content through TnTs. In many instances, we observed TnT-mediated transfer of GFP or M-Orange between cancer and stromal cells. Then, our time-lapse studies confirmed specific movement of dyes along a nanotube suggesting cytoplasmic components transfer from stromal to cancer cells (Figure 3A-B, Additional file 7: Movie 6 and Additional file 8: Movie 7). During phase contrast microscopy analysis, punctual densities were also observed, potentially corresponding to subcellular organelles (Figure 3C-D, Additional file 3: Movie 2). Nanotube mediates transfer of mitochondria between endothelial and endothelial progenitor have been previously described [34]. Therefore, we subsequently investigated the transfer of eGFP-E4^+^ECs mitochondria to cancer cells using the organelle-specific dye Mitotracker. Stained mitochondria could be visualized in the TnTs connecting eGFP-E4^+^ECs to various cancer cells (Figure 4A). Confocal time-lapse imaging demonstrated transfer of mitochondria stained by Mitotracker (Figure 4B and Additional file 9: Movie 8). We also investigated the transfer of eGFP-E4^+^ECs mitochondria to tumor explants. Indeed, we cultured ovarian cancer tumor explants on eGFP-E4^+^ECs feeder stained with Mitotracker-Red. After 48 h of culture, tumor explants were removed and confocal analysis showed uptake of eGFP-E4^+^ECs mitochondria (Figure 4C).

**Figure 5:**
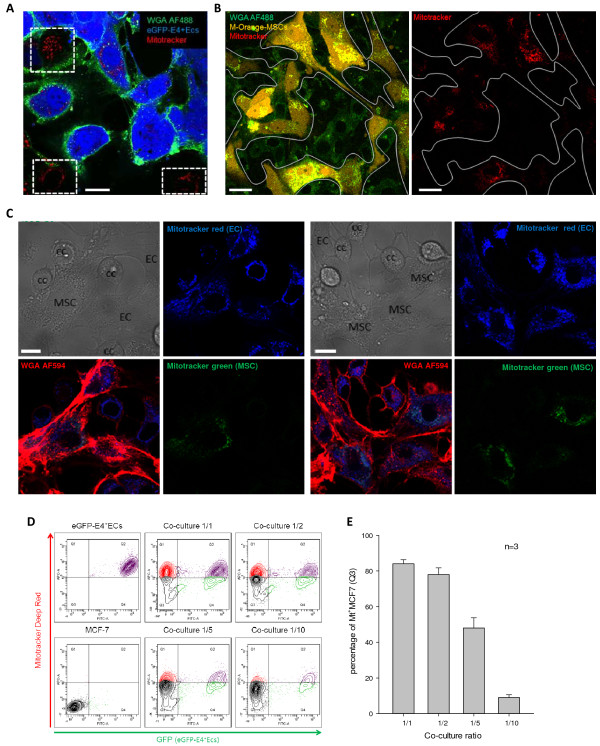
**Preferential exchange of mitochondria from eGFP-E4**^**+**^**ECs to MCF-7. A***,* confocal imaging of co-cultures between MCF7 and eGFP-E4^+^ECs. Prior to co-culture, eGFP-E4^+^ECs were tagged with MitoTracker Deep Red. After 48 hours, co-cultures were stained with WGA AF594 to reveal cell membrane. eGFP-E4^+^ECs mitochondria could be detected in MCF7 (*dotted squares*). *Scale bar: 20 μm*. **B***,* Similar experiments were performed with M-orange-MSCs. No exchange was detected at ratio 1/1. *Scale bar: 20 μm*. **C***,* Tri-culture of MCF7, MSCs (stained with MitoTracker Green) and non-GFP E4^+^ECs (stained with MitoTracker Red) were performed. After 48 hours, cultures were stained with WGA-AF594. E4^+^ECs mitochondria (*blue*) were detected in MCF7 and MSCs, while MSCs mitochondria (*green*) remained restricted to MSCs in this set-up. *Scale bar: 20 μm*. **D***,* Flow cytometry plot of different ratio of co-culture of eGFP-E4^+^ECs/MCF7. Prior to co-culture eGFP-E4^+^ECs were stained with MitoTracker DeepRed. The number of MCF7 up-taking mitochondria from eGFP-E4^+^ECs increased in relation to the number of eGFP-E4^+^ECs. **E**, graphical representations of the flow cytometry findings.

To investigate whether organelle transfer displayed cell specificity MCF7 and eGFP-E4^+^ECs or M-Orange-MSCs co-cultures were established and mitochondria transfer from stromal cells was monitored. TnTs seemed similarly distributed among cell types. However, at a ratio of 1 MCF7 /1 stromal cell, only endothelial cells were able to transfer mitochondria to MCF7s (Figure 5A-B). MSCs mitochondria transfer to cancer cells could only be visualized using an increased ratio (1/10) of MCF7/MSCs (Additional file 1: Figure S4). We then established a tri-culture system (MCF7, MSCs and E4^+^ECs). TnTs were observed between all different cell types, however, in this setting, MCF7 only acquired E4^+^ECs mitochondria (Figure 5C). We could also detect uptake of E4^+^ECs mitochondria by M-Orange-MSCs. Our data provided evidence for a preferential transfer of mitochondria from endothelial cells under our culture conditions.

One of the most common criticisms of such experiment is the ability of the dyes to diffuse. Several findings argued against such bias. The presence of MCF7 cells with different level of Mitotracker after 48 hours of co-culture argued against passive dye transfer. In our tri-culture set-up, the preferential transfer ruled out the possibility of passive diffusion. Indeed it is quite unlikely that dyes would only leak form one cell type. Switching dyes used between E4^+^ECs and MSCs did not modify these findings (data not shown). Flow cytometry evaluation of mitochondria uptake at different ratio of eGFP-E4^+^ECs/MCF7 ranging from 1/1 to 1/10 demonstrated that exchange of mitochondria was related to the number of stromal cells (Figure 5D-E).

Among other mechanisms involved in the cross-talk between cancer et stromal cells, microparticles (MPs) have been involved in the transfer of organelle [35]. We investigated the implication of MPs in the transfer of mitochondria between endothelial and cancer cells. We stained MPs extracted from eGFP-E4^+^ECs using Mitotracker. While cancer cells were able to uptake endothelial MPs, the uptake of mitochondria by MCF7 through MPs was negligible (Additional file 1: Figure S5A-B). Similar results were obtained if endothelial cells were stained before MPs isolation. This indicated that mitochondria transfer from eGFP-E4^+^ECs under our cell culture conditions did not occur through microparticles. We also used a transwell experiment setting to confirm the role of direct cell contact in the transfer of mitochondria. Confocal and flow cytometry analysis could not detect any Mitotracker-Red uptake in the transwell experiments (Additional file 1: Figure S6A-B). In order to rule out the possibility that diffusing Mitotracker into the medium could be adsorbed by the filter of the transwell chambers, we seeded MCF7 on the bottom or the top of the transwell chamber and mitotracker on the other side. Flow cytometry showed constant staining of MCF7 suggesting that passive diffusion could occur through the membrane (Additional file 1: Figure S6C). Finally shaking cultures that do not allow the formation of TnTs were performed [36]. Under these conditions no Mitochondria transfer was observed compare to controls (Additional file 1: Figure S7). These experiments excluded the possibility of massive dye transfer and strongly suggested that TnTs participated to mitochondria transfer.

### Acquisition of mitochondria leads to the emergence of a chemoresistant phenotype

Mitochondria transfer between two cells have been associated to phenotypic modification [37], however a direct functional benefit of TnT mediated material exchange has not yet been demonstrated in the cancer setting. We hypothesized that MCF7 acquiring endothelial mitochondria would display greater chemoresistance. We previously described TnT mediated transfer of P-glycoprotein from resistant to sensitive cells [29]. We first verified that both eGFP-E4^+^ECs and MCF7 did not express *ABCB1* (Data not shown).

**Figure 6:**
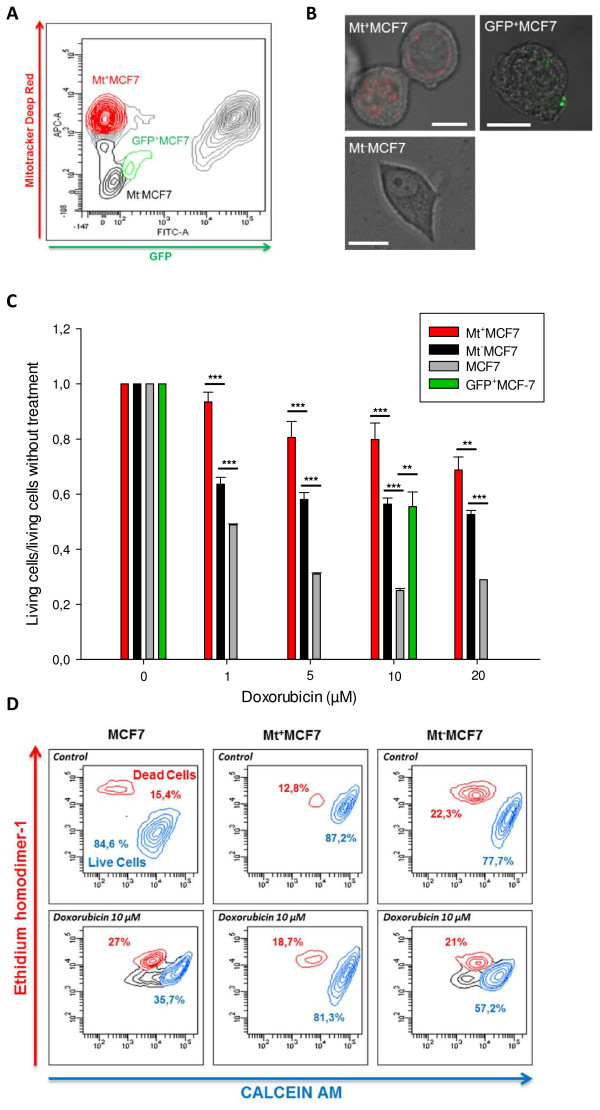
**Transfer of mitochondria from eGFP-E4**^**+**^**ECs to MCF7 confers chemoresistance. A***,* Flow Cytometry Scatter plot showing the gating strategy for definition of the different cell-populations subjected to cell sorting. **B***,* Confocal imaging post-sorting. Mt^+^MCF7 displayed Mitotracker staining (*top left panel*) and GFP^+^MCF7, GFP staining (*top right panel*) however Mt^-^MCF7 did not display Mitotracker nor GFP staining (*bottom panel*). *Scale bar: 10 μm.***C**, MTT assay after doxorubicine treatment was performed on sorted cells. Mt^+^MCF7 population (*red*) displayed significantly increased chemoresistance compared to other control populations. However compared to controls, Mt^-^MCF7 (*black*) or GFP^+^MCF7 (*green*) displayed also chemoresistance (10 μM doxorubicine). (p < 0.05 (*), p < 0.01 (**) or p < 0.001 (***)). **D***,* Cell viability was assessed for Mt^+^MCF7, Mt^-^MCF7 and control MCF7 treated by 10 μM doxorubicine using Live/Dead Invitrogen kit (calcein AM and ethidium homodimer-1). Mt^+^MCF7 displayed chemoresistance. Experiments were performed in triplicate.

To assess functional gain, fluorescence minus one was used to set up the cell-sorting gate as stringent as possible (Figure 6A). Cells having acquired eGFP-E4^+^ECs mitochondria (Mt^+^MCF7, red) and cells did not acquire any mitochondria (Mt^-^MCF7, black) were sorted. Mt^-^MCF7 population allowed us to control for other factors such as endothelial secreted factors or direct cell to cell contact without mitochondria transfer. We finally sorted cells having only acquired cytoplasmic (excluding mitochondria) content represented by the GFP^+^MCF7 population (green). We controlled the purity of our cell sorting by confocal microscopy (Figure 6B) and subjected the different cell populations to a MTT viability assay in presence of doxorubicin; MCF7 cells were used as a control (grey) (Figure 6C). Mt^-^MCF7 and GFP^+^MCF7 displayed chemoresistance (from 1.31 to 1.89 fold compare to MCF7 control). However MCF7 having acquired eGFP-E4^+^ECs mitochondria (Mt^+^MCF7) displayed significantly more chemoresistance compared to MCF7 control (1.91 to 3.19 fold, p < 0.05). These results were confirmed using LiveDead staining (Figure 6D). These experiments indicated that while co-culture and transfer of cytoplasmic content induced chemoresistance, the acquisition of mitochondria significantly increased chemoresistance of MCF 7 to doxorubicin.

## Discussion

In this study, we reported spontaneous TnTs mediated heterocellular exchange between cancer and stromal cells. We demonstrated TnTs formation in different cancer cell types, as well as in two types of stromal cells highly involved in tumor progression. These findings support that TnTs formation are ubiquitous. The occurrence of TnTs in 3D tumor explants is concordant with the presence of TnTs in tumors previously described in human malignant pleural mesothelioma [38]. More interestingly we showed, for the first time TnTs in cancer spheroids, which might be a better model to represent tumor biology and inter-cellular interaction than classical 2D cultures [39].

TnTs between cancer cells and stromal cells had all canonical characteristics previously described [20,24]. Time-lapse studies illustrated that TnTs formation is a common feature and appears to be a dynamic process. We highlighted the primordial role of large cell adhesion domain in the occurrence of TnTs. Thus the formation of TnTs could be consequent to receptor-ligand interaction and exchange of cellular organelles might be dependent of the location and/or nature of initial membrane interaction.

In our different cell culture settings, we demonstrated transfer of cytoplasmic content from stromal to cancer cells. Both MSCs and ECs exchanged cytoplasmic materials with cancer cells without noticeable differences between the cell types. The transfer of cytoplasmic material between similar cell types has been described [30]. Recently some groups have illustrated such phenomena in a heterocellular context [25,37]. We actually observed also transfer of cytoplasmic material form cancer to stromal cells, however the precise significance of this phenomenon is not addressed in this study.

We provided evidence for mitochondrial transfer between stromal and cancer cells through TNTs inducing phenotype modulation. While exchange of cytoplasmic materials appears to happen similarly for MSCs/CCs and E4^+^EC/CCs interactions, we found preferential mitochondria transfer between cancer and endothelial cells in a tri-culture system. We carefully addressed the possibility of leakage and passive diffusion of the dye as follow: (i) we performed cell culture under shaking condition which prevents the formation of TnTs and demonstrated no transfer, (ii) the tri co-culture experiments showed preferential transfer from endothelial toward cancer cells with two different dyes, (iii) in the transwell experiment there was no uptake of mitochondria from the endothelial cells, (iv) Mitotracker used in similar concentration than the one used to stain E4^+^EC was able to cross the membrane of the transwell system and stain cancer cell mitochondria, (v) and finally we were able to follow mitochondria transfer in TnTs by time lapse studies. Using a 3D explant system, we demonstrated that tumor cells of ovarian cancer metastatic nodules could uptake mitochondria from an endothelial cell feeder, suggesting that in dense cellular environments such structures could mediate cell-to-cell communications.

Our analysis of different stromal cell lines pointed out the preferential transfer of mitochondria from endothelial cells compare to MSCs under our culture conditions. This supports the existence of a specific process mediating such exchanges. The specificity of cargoes transferred has already been illustrated without a precise mechanism, however structural components, specific signaling pathways and/or culture conditions may all play a role [40].

Mitochondria transfer has already been described in different homo- and heterocellular models such as endothelial progenitors and cardiomyocytes [25], astrocytes [41], primary human renal epithelial cells [42], vascular smooth cells and MSCs [37], human retinal pigment epithelial cells [43] and between human malignant pleural mesothelioma cells [38]. Among these studies, only one associated a functional gain via TnT dependent mitochondria exchange. Indeed, Vallabhaneni et al. demonstrated that vascular smooth muscle cells initiate proliferation of mesenchymal stem cells through exchange of mitochondria via TnTs in cocultures [37]. Here, we illustrated the phenotypic advantage of mitochondria uptake. Using a cell sorting strategy controlling for secreted factors, cytoplasmic transfer or cell-to-cell contact, we showed that cells acquiring mitochondria displayed significantly chemoresistance. Functional benefits (survival and resistance to stress) from acquisition of mitochondria have been documented. Spees et al. showed that active transfers of mitochondria could rescue aerobic respiration in cells with dysfunctional mitochondria [44]. They indicated that genetic defects in mitochondria DNA could be rescued by uptake of normally functioning mitochondria from wild-type cells ex vivo. Oliva et al. described the role of increased mitochondrial activity resulting in decreased reactive oxygen species production and in acquisition of chemoresistance in gliomas [45]. Our study is the first reporting chemoresistance linked to mitochondria uptake in a heterocellular context.

Chemotherapy mostly aims at directing specifically cancer cells into apoptosis. Apoptosis is a complex multi-step mechanism. However mitochondrial membrane permeability is a primordial step of apoptosis. Classical anticancer agents such as etoposide, doxorubicin, cisplatin, or paclitaxel (Taxol) cause indirect mitochondrial permeability as they trigger apoptosis by inducing p53 expression; activation of the ceramide/GD3 pathway; by inducing the CD95/CD95L ligand system, affecting Bcl-2- like proteins; and/or by compromising the redox or energy balance [46,47]. Proteins of the Bcl family mediate stabilization of mitochondrial membrane thus preventing apoptosis. Our discovery of mitochondrial transfer from the microenvironment to tumor cells raises questions about tumor cell response to DNA-damaging anticancer drugs.

We acknowledge that other mechanisms might be implicated in conferring chemoresistance such as transfer of other cytoplasmic components (proteins, genetic material), or transcriptome modifications and this will be addressed in further studies.

The presence of TnTs in vivo has been clearly established in the context of developmental biology (between dendritic cell in the mouse cornea [22]; Epithelial Cells in Live Drosophila Wing Imaginal Discs [48]) or neoplasia (mesothelioma tumor biopsies [38]). Their role in different cellular functions ranging from transmission of prions [28,49,50] or virus particles [27,51], electrical coupling [33,52] cell death signaling [53] to chemoresistance acquisition through P-Glycoprotein transfer [29], points out the importance of such physiological process in multicellular organisms.

Many studies point out the importance of cellular contact in mediating tumor phenotype modulation [17,29,54,55]. There are evidences in the literature that relative cellular position in a multicellular context modulate phenotype. Indeed we have previously illustrated that the Hematopoietic Stem cells reside next to a sinusoidal endothelial niche in the bone marrow [56]. While many local factors (cytokines, membrane bound) certainly play a role, contact mediated phenotypic transfer has a role in specific contexts. The chemoresistance provided by the transfer of mitochondria can lead to the emergence of a residual microscopic disease through dynamic phenotype modulation. This emphasize the role of a metastatic niche where the endothelial-tumor cell contact dependant interaction, among other mechanisms, could protect few cancer cells from chemotherapeutic cytotoxicity [57]. Our co-culture experiment and differential sorting strategy revealed the occurrence of phenotypic heterogeneity relying on the interaction between cancer cells and stromal cells. There are many aspects that need to be investigated: understanding the mechanisms underlying (i) the specificity of cell exchange (ii) the constitution of TNTs post-adhesion and (iii) the role of newly acquired mitochondria in the phenotype modification of cancer cells as targeting mitochondria might be a valuable approach in cancer treatments [58].

All together our comprehensive approach points out the importance of TnTs in cross-talk between cancer cells and stromal cells and underlines specificity of such a physiological process. The role of TnTs mediated communication needs to be considered in the context of other cell-to-cell interactions (secreted factors, microparticles, direct cell contact). Tailoring the importance of such mechanisms will lead to a better understanding of tumor dynamics and to design new therapeutic approaches.

### Ethics

IRB was obtained at Hamad Medical Corporation.

## Competing interests

The authors declare that they have no competing interests.

## Authors’ contributions

Conception and design: JP and AR. Acquisition of data: JP, HAT, PG, MM, NAK and AJ. Analysis and interpretation of the data: JP, MM, LG, FLF and AR. Manuscript preparation: JP and AR wrote the manuscript. Manuscript reviewing: JP, BSG, SR, FLF and AR. All authors read and approved the final manuscript.

## Supplementary Material

Additional file 1: Figure S1 Intracellular bridges are present in monocultures of cancer cells, endothelial cells and MSCs. Confocal imaging of cancer cells (**A**) or eGFP-E4^+^ECs (**B**) or M-orange-MSCs (**C**) stained with WGA AF594. Homo-cellular TnTs connections can be observed between the different cells used in this study **A**, *Scale bar: 10 μm*. **B**, *Scale bar: 20 μm*. **C**, *Scale bar: 10 μm*. **Figure S2**. TnTs displayed canonical features. Alexa Fluor 594 conjugated-WGA-stained cells were analyzed by live cell confocal microscopy. Observed TNTs were submitted to Z-stack analysis (**A**-**B**). TNTs were not in contact with the substratum. **C**, Surface rendering (Imaris) illustrating the independency of the TNTs from the substratum (MCF7). **D**-**E**, TnTs have a diameter smaller than 0.5 μm and a length of up to several cell diameters. *Scale bar: 20 μm*. **F**, Fixed cells were stained with WGA, DAPI and AlexaFluor 647 conjugated-phalloidin. Merged images display co-localization of WGA and TRITC-phalloidin staining, indicating that F-actin is a component of TNTs. *Scale bar: 10 μm*. **Figure S3**. TnTs connect cells in tumor explants and within spheroids. **A**, Ovarian cancer explants were cultured for 10 days. TnT-like structures were detected in the migrating cells of the explant. Cytoplasmic materials could be observed inside the TnTs-like structure (arrows). *Scale bar: 20 μm*. **B**-**C**, OVCAR3 or MCF7 were grown in spheroid with eGFP-E4^+^ECs in 3D media on low adherent plates. After 3 days, spheroids were stained with WGA-AF594. TnTs-like structures were detected connecting cells within spheroid structures. (PDF 776 kb) *Scale bar: 10 μm*. **Figure S4**. Mitochondria are transferred through TnTs between MSCs and Cancer cells. **A**-**C**, Co-culture of M-Orange-MSCs stained with MitoTracker DeepRed and MCF7 at a ratio of 10/1 were performed. Transfer of mitochondria between MSCs and cancer cells could be observed. *Scale bar: 20 μm.***C**, Cancer Cells with different amount of mitochondria uptake could be observed suggesting active dye transfer. *Scale bar: 20 μm.***Figure S5**. Microparticles do not mediate mitochondria transfer. Microparticles were extracted from eGFP-E4^+^ECs and stained with WGA AF594 (**A**) or MitoTracker Red (**B**). MCF7 were incubated with the stained microparticles for 24 hours. MCF7 were able to uptake microparticles from eGFP-E4^+^ECs (**B**) there was, however, negligible microparticle-mediated passage of mitochondria from eGFP-E4^+^ECs to MCF7. *Scale bar: 10 μm*. **Figure S6**. Transwell experiments. **A**-**B**, eGFP-E4^+^ECs were stained with MitoTracker DeepRed. MCF7 and stained eGFP-E4^+^ECs were co-incubated in a transwell system. No mitochondria exchange could be detected neither by confocal microscopy (**A**) nor flow cytometry (**B**). *Scale bar: 20 μm*. **C**, MCF7 cells were stained by MitoTracker DeepRed through a transwell membrane compare to controls (*middle plot:* MCF7 plated on the upper side of the transwell, *right plot*: at the bottom of the membrane). Transwell experiment demonstrating the ability of the Mitotracker dye to stain the MCF7 cells through the transwell system in different settings. **Figure S7**. Shaking culture abrogates the transfer of mitochondria from Endothelial to cancer cells. MCF7 (*grey*) were co-culure in normal or shaking conditions with eGFP-E4 + ECs (*black*) stained with Mitotracker DeepRed. While there was a gain of Mitotracker by MCF7 in control set-up (*red*) no transfer could be observed during shaking (*dash red*). Click here for file

Additional file 2: Movie 1 Surface rendering of MCF7 cells interconnected by TnTs. Surface rendering of Z-stack confocal imaging of MCF7 with IMARIS software. Click here for file

Additional file 3: Movie 2 TnTs appear spontaneously in co-culture between MCF7 and MSC. 6 hours after plating, co-culture of MCF7 and MSCs were imaged by time-lapse microscopy. The movie sequence was acquired during 24 hours. Click here for file

Additional file 4: Movie 3 Formation of TnTs after large membrane adhesion between ovarian cancer cells. SKOV3 cells were analyzed by confocal microscopy time-lapse imaging. Cells were stained with WGA-AF594 to reveal the membrane (*red*). The movie sequence represents 1 hour of acquisition. Formation of TnTs after large membrane adhesion are observed. Click here for file

Additional file 5: Movie 4 Formation of TnTs after large membrane interaction between eGFP-E4 + ECs and breast cancer cells. Co-culture of MDA and eGFP-E4^+^ECs (*green*) cells was stained with WGA-AF594 to reveal the membrane (*red*). Stained co-culture was analyzed by confocal microscopy time-lapse imaging. The movie sequence represents 1 hour of acquisition. Click here for file

Additional file 6: Movie 5 Formation of TnTs after membrane interaction between eGFP-E4 + ECs. eGFP-E4^+^ECs cells (*green*) were stained with WGA-AF594 to reveal the membrane (*red*) and analyzed by confocal microscopy time-lapse imaging. The movie sequence represents 40 minutes of acquisition. Click here for file

Additional file 7: Movie 6 TnT-mediated cytoplasmic components transfer from eGFP-E4 + ECs to ovarian cancer cells. Co-culture of eGFP-E4 + ECs (*green*) and OVCAR3 were stained with WGA AF594 (*red*) to reveal the membrane and analyzed by confocal microscopy time-lapse imaging. TnT-mediated transfer of WGA stained cytoplasmic content was observed (*arrow*). The movie sequence represents 1 hour of acquisition. Click here for file

Additional file 8: Movie 7 GFP transfer from eGFP-E4 + ECs to MCF7 through TnTs. Co-culture of eGFP-E4 + ECs (*green*) and MCF7 were stained with WGA AF594 (*red*) to reveal the membrane and analyzed by confocal microscopy time-lapse imaging. GFP transfer through TnTs was visualized (*arrow*). The movie sequence represents 35 minutes of acquisition. Click here for file

Additional file 9: Movie 8 Time-lapse imaging of Mitochondria transfer through TnTs. eGFP-E4 + ECs were stained with Mitotracker DeepRed prior to the co-culture (*green*). Co-culture of eGFP-E4 + ECs and MCF7 was stained with WGA AF594 to reveal the membrane (*red*). Co-culture was analyzed by confocal microscopy time-lapse imaging. Movie is showing the mitochondria staining (*green*) and the WGA staining (*red*) in the same time. Time-lapse imaging shows transfer of mitochondria among cytoplasmic content through a TnT. The movie sequence represents 2 hours of acquisition. Click here for file
