# Supplementary material for: Preferential transfer of mitochondria from endothelial to cancer cells through tunneling nanotubes modulates chemoresistance
Source: J Transl Med. 2013 Apr 10;11:94. doi: 10.1186/1479-5876-11-94 (PMC3668949; doi:10.1186/1479-5876-11-94)

Supplementary Figure 1.

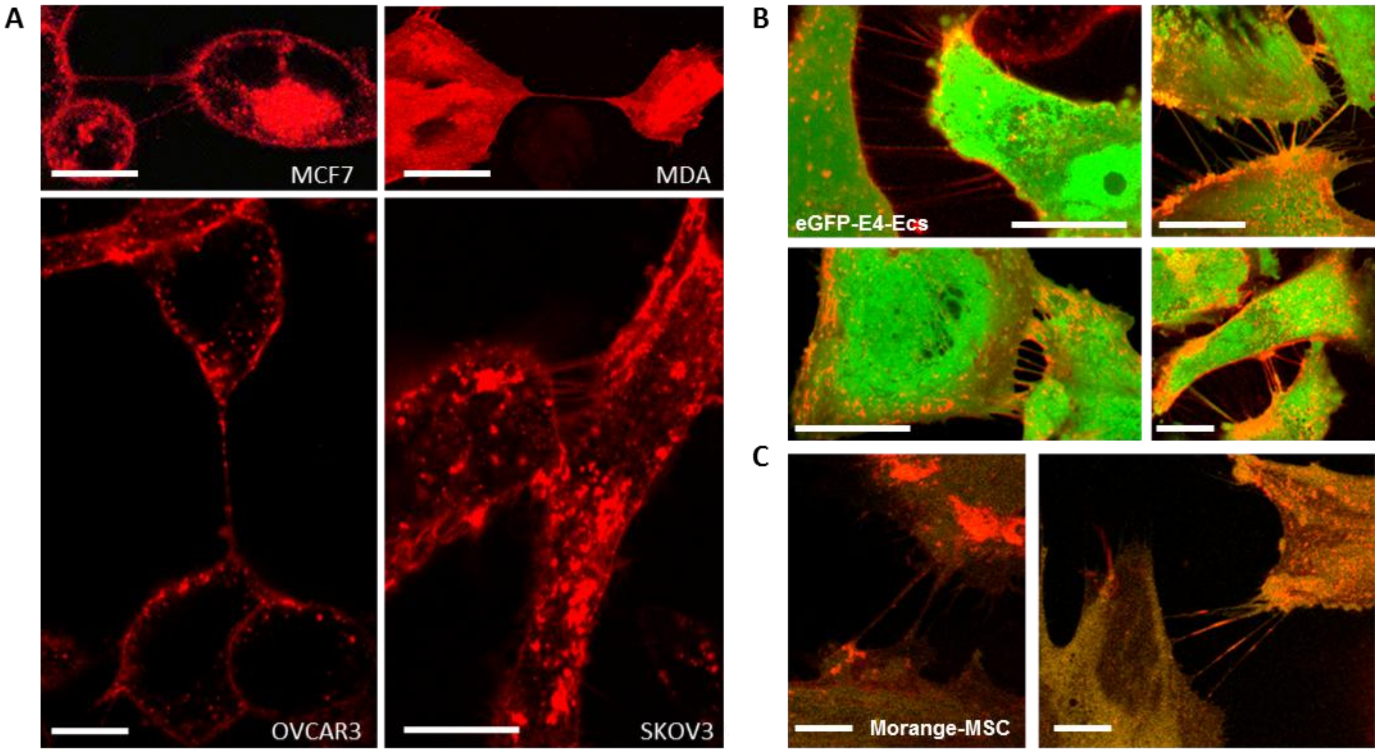

Supplementary Figure 2.

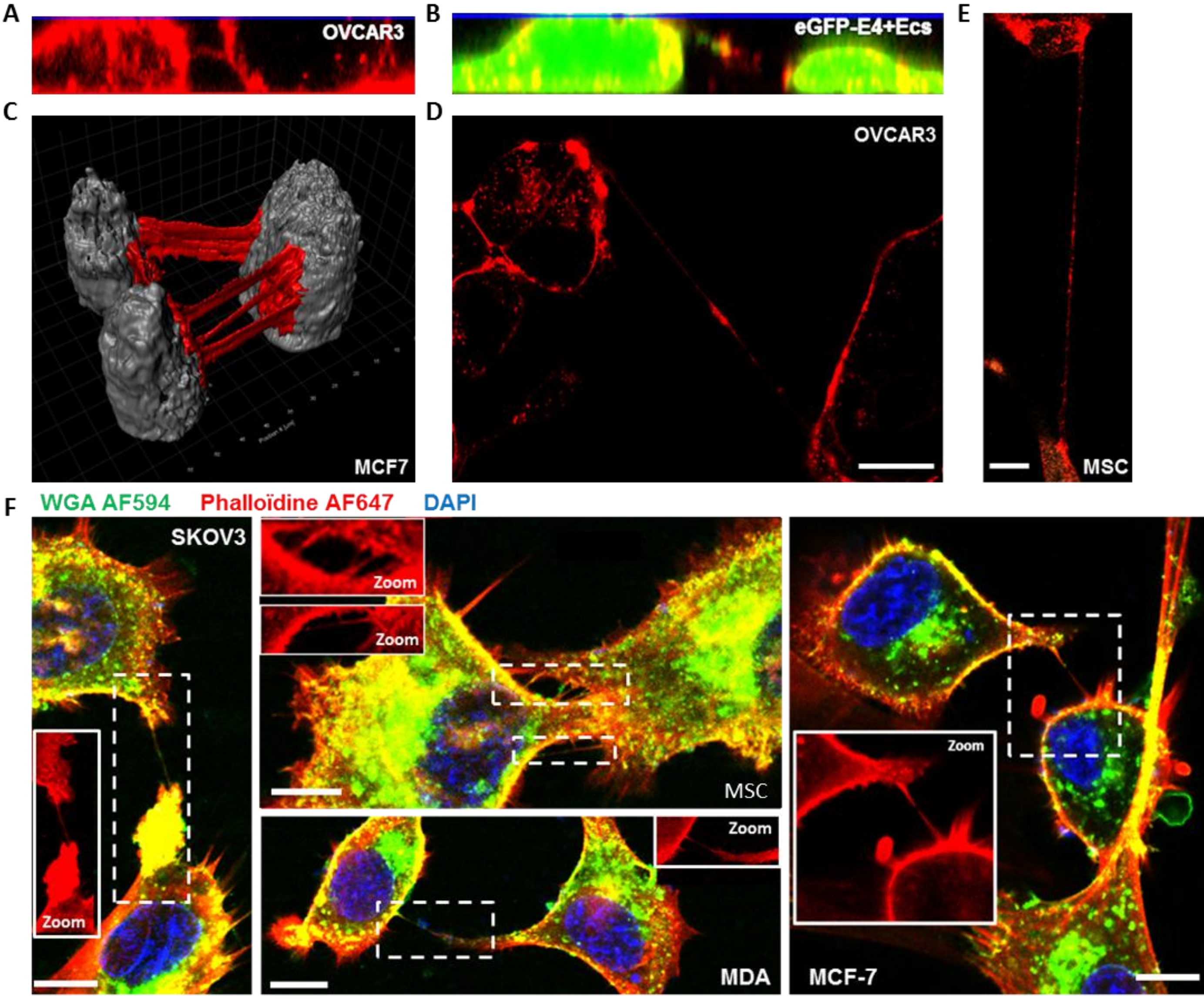

Supplementary Figure 3.

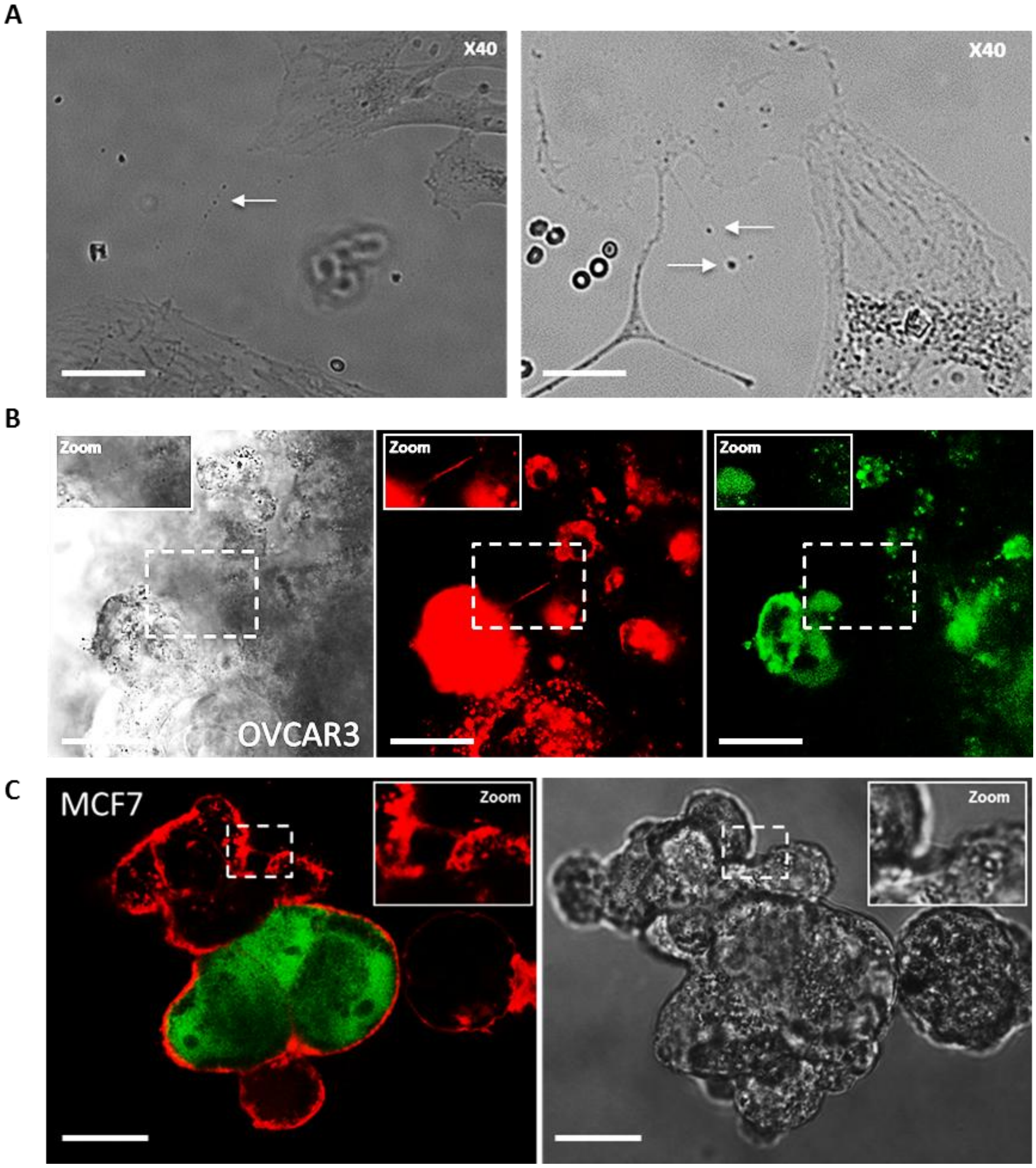

Supplementary Figure 4.

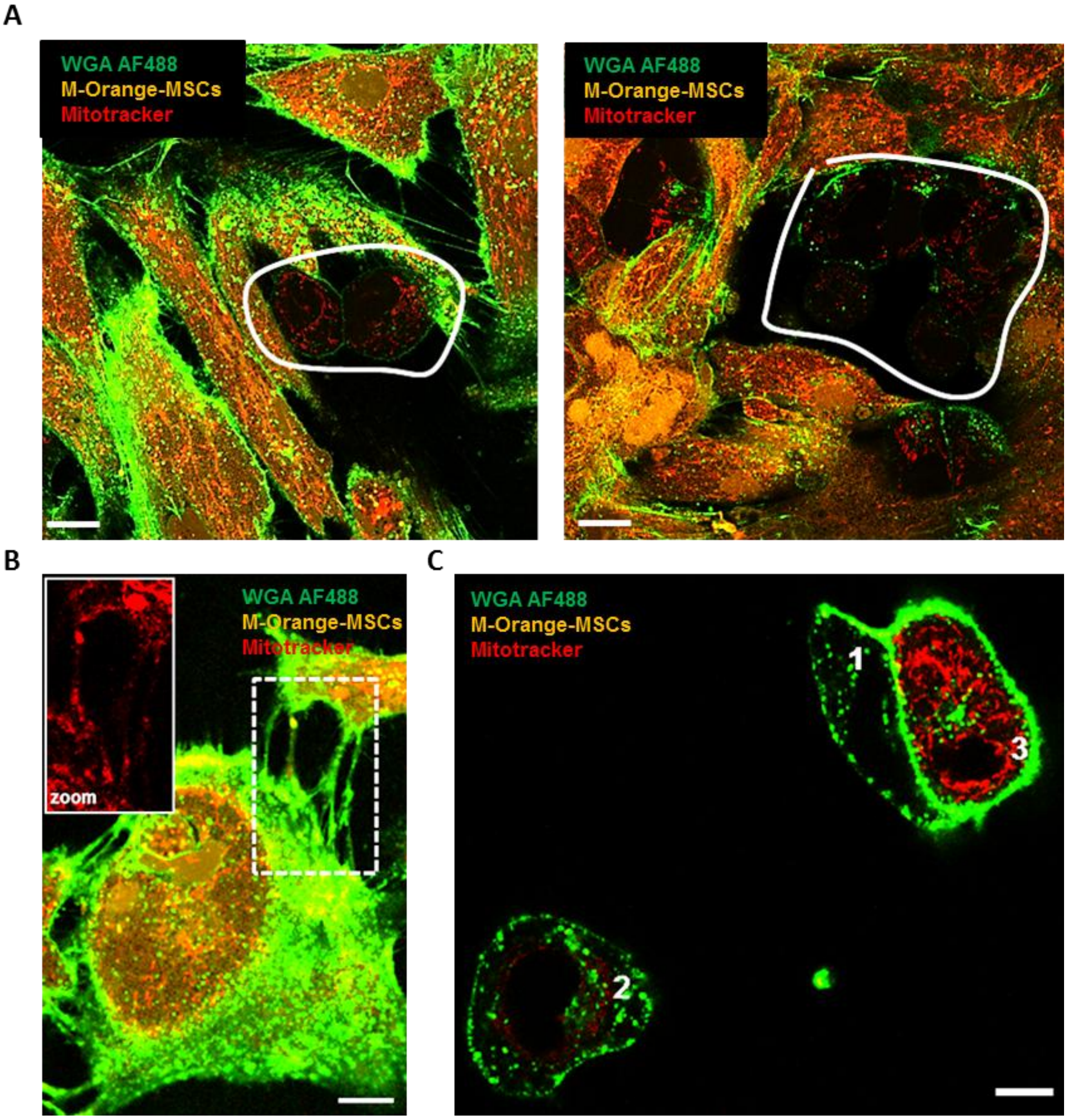

Supplementary Figure 5.

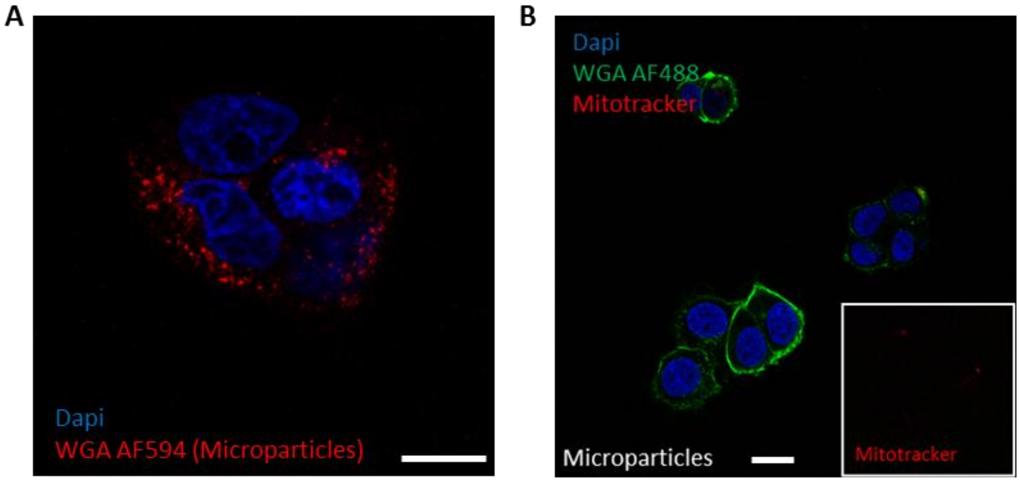

Supplementary Figure 6.

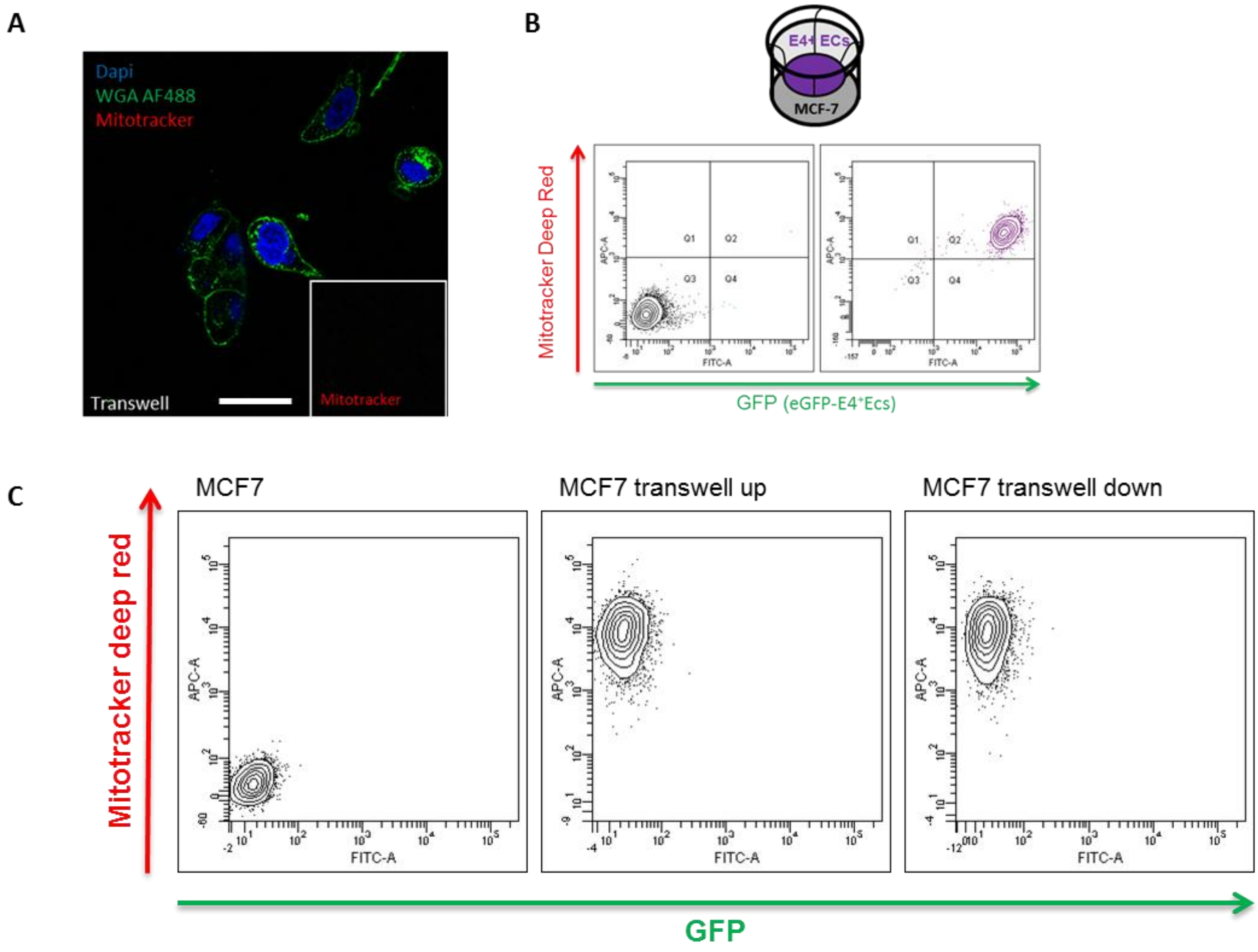

Supplementary Figure 7.

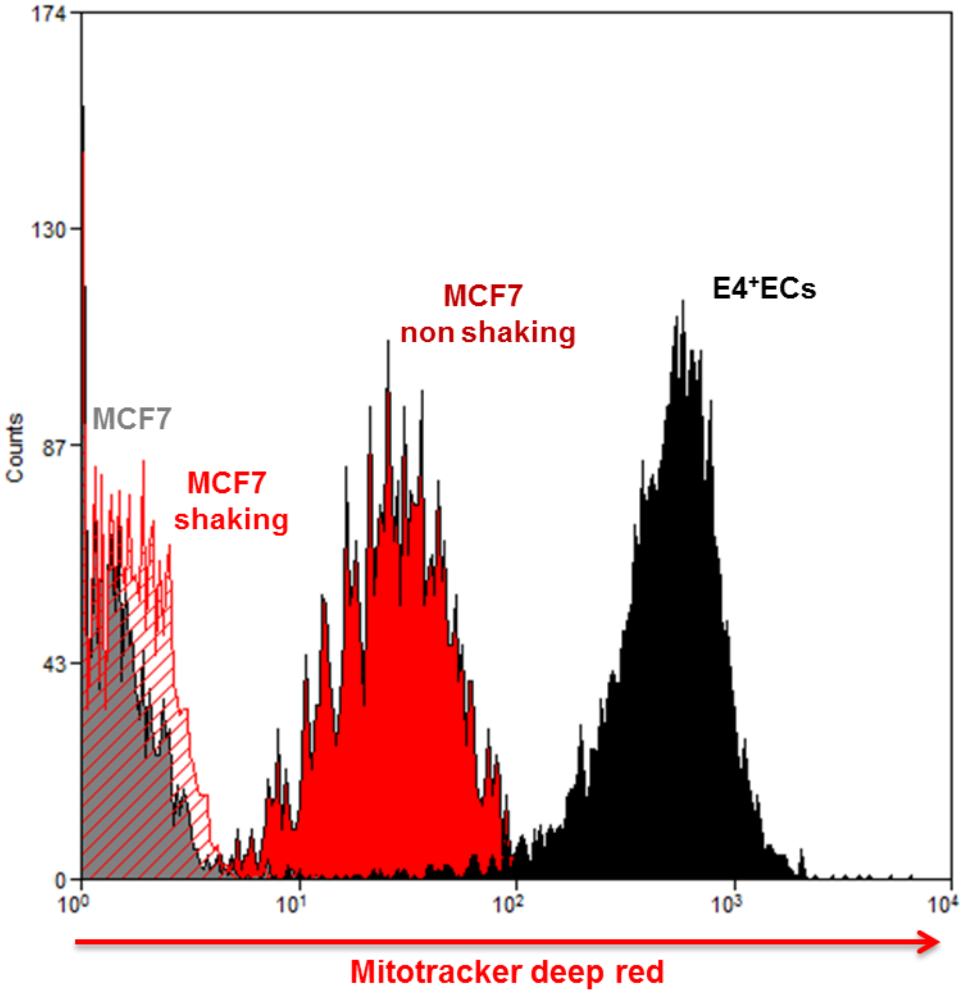

Supplement: Additional file 1: Figure S1 — Intracellular bridges are present in monocultures of cancer cells, endothelial cells and MSCs. Confocal imaging of cancer cells (A) or eGFP-E4+ECs (B) or M-orange-MSCs (C) stained with WGA AF594. Homo-cellular TnTs connections can be observed between the different cells used in this study A, Scale bar: 10 μm. B, Scale bar: 20 μm. C, Scale bar: 10 μm. Figure S2. TnTs displayed canonical features. Alexa Fluor 594 conjugated-WGA-stained cells were analyzed by live cell confocal microscopy. Observed TNTs were submitted to Z-stack analysis (A-B). TNTs were not in contact with the substratum. C, Surface rendering (Imaris) illustrating the independency of the TNTs from the substratum (MCF7). D-E, TnTs have a diameter smaller than 0.5 μm and a length of up to several cell diameters. Scale bar: 20 μm. F, Fixed cells were stained with WGA, DAPI and AlexaFluor 647 conjugated-phalloidin. Merged images display co-localization of WGA and TRITC-phalloidin staining, indicating that F-actin is a component of TNTs. Scale bar: 10 μm. Figure S3. TnTs connect cells in tumor explants and within spheroids. A, Ovarian cancer explants were cultured for 10 days. TnT-like structures were detected in the migrating cells of the explant. Cytoplasmic materials could be observed inside the TnTs-like structure (arrows). Scale bar: 20 μm. B-C, OVCAR3 or MCF7 were grown in spheroid with eGFP-E4+ECs in 3D media on low adherent plates. After 3 days, spheroids were stained with WGA-AF594. TnTs-like structures were detected connecting cells within spheroid structures. (PDF 776 kb) Scale bar: 10 μm. Figure S4. Mitochondria are transferred through TnTs between MSCs and Cancer cells. A-C, Co-culture of M-Orange-MSCs stained with MitoTracker DeepRed and MCF7 at a ratio of 10/1 were performed. Transfer of mitochondria between MSCs and cancer cells could be observed. Scale bar: 20 μm.C, Cancer Cells with different amount of mitochondria uptake could be observed suggesting active dye transfer. Scale bar: 20 μm. [file 1479-5876-11-94-S1.pdf]
